# Glutamine Modulates mVOC Biosynthesis in *Streptomyces alboflavus* Through a *gluR*-Dependent Signaling Pathway and Enhances Its Inhibitory Activity Against *Aspergillus flavus*

**DOI:** 10.3390/foods15020228

**Published:** 2026-01-08

**Authors:** Wangqiang Li, Mingguan Yang, Zehua Dong, Tong Liu, Xiuyu Liu, Dan Liu, Chengfang Ding, Laifeng Lu, Wentao Ding, Zhenjing Li, Huanhuan Liu, Zhifang Wang, Qingbin Guo, Changlu Wang

**Affiliations:** 1 State Key Laboratory of Food Nutrition and Safety, School of Food Science and Engineering, Tianjin University of Science & Technology, Tianjin 300457, China; liwangqiang1993@gmail.com (W.L.);; 2 Key Laboratory of Industrial Fermentation Microbiology, Tianjin Key Laboratory of Industrial Microbiology, Ministry of Education, Tianjin University of Science and Technology, Tianjin 300457, China; 3 College of Food Science and Engineering, Qilu University of Technology (Shandong Academy of Sciences), Jinan 250353, China; 4 Analytical & Testing Center, Tianjin Polytechnic University, Tianjin 300387, China

**Keywords:** biocontrol, CRISPR/Cas9 genome editing, headspace solid-phase microextraction (HS-SPME), nitrogen metabolism, secondary metabolism

## Abstract

*Aspergillus flavus* and its aflatoxins pose serious threats to human and animal health, negatively affecting agricultural productivity and the global economy. Although chemical preservatives are widely used, their effectiveness remains limited by increased fungal resistance and environmental concerns, highlighting the need for sustainable alternatives. Microbial volatile organic compounds (mVOCs) represent a promising biocontrol strategy. Here, we investigate how glutamine regulates mVOC biosynthesis in *Streptomyces alboflavus* TD-1 and enhances its antifungal activity against *A. flavus*. Antifungal assays showed that supplementation with 40 mM glutamine significantly enhanced inhibitory activity, leading to 69.0% inhibition of conidial germination and 64.5% inhibition of mycelial biomass. Transcriptome profiling identified 283 differentially expressed genes, including the two-component system regulator *gluR*, which was strongly upregulated. CRISPR/Cas9-mediated disruption of *gluR* confirmed its regulatory role. Specifically, the mutant strain produced reduced levels of antifungal mVOCs, such as dimethyl trisulfide and *o*-anisidine, and exhibited diminished inhibition of *A. flavus*. Collectively, these findings demonstrate that exogenous glutamine enhances the mVOC-mediated suppression of *A. flavus* by *S. alboflavus* TD-1 through nutrient-sensing and transcriptional regulation of volatile biosynthesis. Although aflatoxin levels were not quantified in this study, the enhanced growth inhibition and the identified mVOC shifts provide a mechanistic basis for future studies that directly quantify aflatoxin production under storage-relevant conditions.

## 1. Introduction

*Aspergillus flavus* is a saprophytic filamentous fungus that is widespread in the environment, and is a pathogen commonly found in food and agricultural products [1,2]. *A. flavus* produces aflatoxins (AFs; B1, B2, G1, G2, M1, and M2), which are among the most toxic and carcinogenic compounds known to humans [3]. The effects of aflatoxins in animals include nephrotoxicity, hepatotoxicity, mutagenesis, teratogenesis, and immunosuppression [4]. Aflatoxin contamination is a major global public health concern despite the availability of multiple control strategies [5,6].

Such contamination is especially prevalent in nuts, grains, and dried fruits [7]. In the context of food processing, the widespread use of chemical fungicides in combating *A. flavus* and aflatoxin contamination has raised concerns due to their negative impacts on human health, including food safety, environmental pollution, plant toxicity, and chemical resistance [8,9,10]. By contrast, biological control relies on bioactive secondary metabolites from plants and microbes, offering an efficient, safe, and environmentally friendly alternative that has been shown to effectively suppress fungal pathogens [11,12,13].

mVOCs represent an environmentally friendly alternative to chemical fungicides. Being naturally volatile, carbon-based compounds, mVOCs disperse easily under ambient conditions, making them particularly suitable for agricultural applications [14,15,16,17,18]. These agents demonstrate broad-spectrum efficacy against a range of pathogens, addressing both increasing fungal resistance and environmental concerns associated with conventional fungicides [19]. Extensive research has validated the antimicrobial properties of mVOCs [20]. For instance, *Muscodor albus* and *Streptomyces* sp.—prolific producers of these compounds—have demonstrated substantial activities that prevent microbial spoilage and contamination in crops such as pistachios, dried figs, Indian senna, corn grains, and peanuts [21,22,23,24]. Among these mVOCs, dimethyl trisulfide has been reported to inhibit *A. flavus* growth and reduce aflatoxin production, which is critical for ensuring food safety and quality [25,26]. Furthermore, Yang et al. (2019) demonstrated that dimethyl trisulfide produced by *S. alboflavus* TD-1 effectively suppressed *A. flavus*, supporting its practical use as a biopesticide and confirming its role in biological control strategies [25]. This research highlights the potential of mVOCs for food safety management and indicates that mVOC-based inhibition could be explored as a low-residue strategy for controlling *A. flavus* during postharvest handling and grain storage, potentially reducing dependence on chemical preservatives [20,27].

Given the central role of *Streptomyces* sp. in mVOC production, elucidating their regulatory mechanisms is essential for improving biocontrol strategies. *Streptomyces* sp. are Gram-positive, filamentous soil bacteria characterized by high genomic guanine–cytosine (GC) content. They exhibit complex life cycles and produce a wide range of secondary metabolites with applications in medicine, industry, and agriculture [4,28,29]. *Streptomyces* sp. possess two-component systems (TCSs) that play a crucial role in regulating both primary and secondary metabolism [30]. These systems encompass *mtrA*/*B*, *ragK*/*G*, *phoP*/*R*, *glnR*, and *cssR*/*S*. Additionally, they influence the synthesis of bioactive molecules [31,32,33,34,35].

Beyond serving as a nitrogen donor in primary metabolism, glutamine also functions as a nutritional supplement that can modulate microbial physiology [36]. In microorganisms such as *Streptomyces*, glutamine availability influences nitrogen assimilation, amino acid biosynthesis, and secondary metabolism through regulatory systems including *glnR* and *gluR*/*K* [37]. As a signaling molecule, glutamine can activate or repress gene networks related to the synthesis of bioactive compounds, including antibiotics and volatile metabolites. Supplementation with glutamine has been reported to enhance growth, optimize metabolic flux toward secondary metabolites, and improve biocontrol efficacy against plant pathogens [38]. Given these properties, glutamine holds potential as a simple, cost-effective additive to boost the antifungal activity of microbial agents in agricultural applications [39].

This study investigated how exogenous glutamine supplementation influences mVOC biosynthesis in *S. alboflavus* TD-1 and enhances its antifungal activity against *A. flavus*, with a particular focus on the GluR-mediated regulatory pathway. The findings provide new insights into glutamine as a metabolic modulator for improving mVOC-based biocontrol for suppressing *A. flavus* growth. This is consistent with the increasing need for natural and eco-friendly solutions in the food industry, highlighting the significance of our research within the wider scope of food microbiology and public health.

## 2. Materials and Methods

### 2.1. Microorganisms

*S. alboflavus* TD-1 was originally isolated from soil in Tianjin, China, and identified as described previously (GenBank accession No. JX915780) [40]. This strain was maintained on Gause’s synthetic agar slants with 20% glycerol and stored at −80 °C for long-term preservation. Before experiments, cultures were grown on fresh Gause’s synthetic agar slants at 28 °C for 5 days. Mature colonies were suspended in 10 mL of sterile water containing 0.01% (*v*/*v*) Tween 80, gently scraped with a sterile cotton swab, and centrifuged at low speed. For mVOC production, 10 g of autoclaved wheat bran was placed in 250 mL conical flasks, inoculated with 2 mL of spore suspension (1 × 10^6^ sp-ore/mL), and incubated statically at 28 °C for 8 days.

*A. flavus* (CICC 2219), originally isolated from soy sauce residue, was obtained from the China Center of Industrial Culture Collection (Beijing, China) and cultured on PDA plates at 28 °C for 3 days. The culture was maintained at 4 °C. Conidia were harvested in 10 mL of sterile 0.05% Tween 80, filtered through two layers of autoclaved gauze to remove mycelia, and adjusted to 1 × 10^6^ conidia/mL with a hemocytometer.

### 2.2. Effects of S. alboflavus TD-1 mVOCs on Conidial Germination and Mycelial Biomass of A. flavus

The antifungal activity of *S. alboflavus* TD-1 metabolites against *A. flavus* was assessed using a dual-Petri dish method [41]. Two 60 mm Petri dishes were placed face-to-face and sealed with two layers of Parafilm. The lower dish contained *S. alboflavus* TD-1 grown on Gause’s synthetic agar supplemented with 0, 20, 40, 60, 80, or 100 mM glutamine. In this study, antifungal efficacy was evaluated using growth-related endpoints (conidial germination and dry mycelial biomass). Aflatoxin production was not quantified under mVOC exposure in this study.

For the conidial germination assay, the upper dish contained 5 mL PDA inoculated with 100 μL of an *A. flavus* conidial suspension (1 × 10^6^ conidia/mL). After incubation at 28 °C for 3 days, inhibitory effects were evaluated by counting conidia to determine the inhibition of conidial germination.

To determine the dry mycelial biomass, the upper dish contained 5 mL PDA inoculated with a 6 mm agar plug of *A. flavus*. After incubation under the same conditions (28 °C, 3 days), the mycelial mat was harvested and dried at 60 °C to constant weight. All treatments were performed in triplicate. The inhibition rate of conidial germination was calculated as follows:

Inhibition (%) = (*A_c_* − *A_t_*)/*A_c_* × 100%

where *A_c_* is the number of conidia in the control, and *A_t_* is the number of conidia during testing.

### 2.3. mVOCs Extraction and GC-MS Analysis

To analyze mVOCs associated with antifungal activity, *S. alboflavus* TD-1 was cultured on Gause’s synthetic agar supplemented with 0, 20, 40, 60, 80, or 100 mM glutamine in the bottom plate of a sealed dual-Petri dish system. Cultures were incubated statically at 28 °C for 8 days. mVOCs were extracted, separated, and identified, and their differential abundance (log2 (EG/CG)) was determined using headspace solid-phase microextraction (HS-SPME) coupled with gas chromatography–mass spectrometry (GC-MS).

A 2 cm SPME fiber (50/30 μm DVB/CAR/PDMS, Supelco, Bellefonte, PA, USA) was exposed to the headspace for 30 min at 45 °C to adsorb volatiles. The fiber was desorbed in the injection port of a GC/MSQP 2010 Ultra (Shimadzu, Kyoto, Japan) at 250 °C for 2 min in splitless mode. A VF-5MS capillary column (30 m × 0.25 mm i.d., 0.25 μm film; Varian Inc., Palo Alto, CA, USA) was used for separation and identification of volatiles [42]. Helium was used as the carrier gas at a constant flow of 1 mL/min. The following oven program was used: 40 °C for 3 min, increased to 150 °C at 4 °C/min, then ramped to 250 °C at 8 °C/min, and held for 6 min. The mass spectrometer was operated in electron impact (EI) mode at 70 eV, scanning from *m*/*z* 43 to 500. Compounds were identified by comparing mass spectra with the NIST 2011 Mass Spectral Library and further confirmed by comparing calculated retention indices (RI), determined using C7–C30 n-alkanes under identical conditions, with values reported in the literature.

### 2.4. Total RNA Isolation, RNA Sequencing, and Analysis

Mycelia were collected by filtering cultures through sterile gauze, washed with sterile PBS, and blotted dry on sterile filter paper. The harvested mycelium was immediately frozen in liquid nitrogen and stored at –80 °C until RNA extraction. Total RNA was extracted using Trizol Reagent (Invitrogen Life Technologies, Carlsbad, CA, USA) according to the manufacturer’s instructions, and RNA quality and concentration were assessed with an Agilent 2100 Bioanalyzer (Agilent Technologies, Palo Alto, CA, USA) [43].

Ribosomal RNA was removed using the Ribo-off rRNA Depletion Kit (Vazyme Biotech, Nanjing, China). Strand-specific, indexed cDNA libraries were prepared using the SMARTer Stranded RNA-Seq Kit (TaKaRa Bio USA, Mountain View, CA, USA). Libraries were sequenced on an Illumina platform (Illumina, San Diego, CA, USA). Subsequent quality control, de novo assembly (Trinity v2.0.6), and annotation were performed as described by Grabherr et al., using BLASTx (NCBI BLAST+ v2.15.0) against the NR database and mapping to GO, KEGG, Swiss-Prot, and eggNOG databases [44].

Gene expression levels were normalized as log_2_-transformed FPKM values and analyzed using RSEM v1.3.0. Differential expression analysis was conducted using DESeq2 (v1.20.0, Bioconductor, New York, NY, USA) [45]. Genes with a fold change >2 and adjusted *p*-value < 0.05 were defined as differentially expressed (DEGs). GO and KEGG pathway enrichment analyses were performed to uncover the functions of DEGs.

### 2.5. qRT-PCR Analysis

To validate the RNA-Seq data, seven genes involved in mVOC biosynthesis were randomly selected for qRT-PCR analysis. Reactions were performed in a 25 µL volume containing 1 µL cDNA (<100 ng) on an Mx3000p instrument (Stratagene, La Jolla, CA, USA). Gene expression was normalized to 16S rRNA, and relative expression was calculated using the 2^−ΔΔCT^ method. Primer specificity was verified with melting curve analysis, and all reactions displayed a single sharp peak. All reactions included three biological and three technical replicates. Statistical significance was assessed using Student’s *t*-test (*p* < 0.05). Primers and reference genes are listed in Table 1.

### 2.6. CRISPR/Cas9-Mediated Deletion of gluR in S. alboflavus TD-1

*E. coli* DH5α was used as the host strain for cloning and plasmid construction. *E. coli* ET12567/pUZ8002 was used as the nonmethylating plasmid donor for intergeneric conjugation with *S. alboflavus* TD-1. The replicative vector pCRISPomyces-2, which harbors an apramycin resistance marker, was used as the CRISPR/Cas9 genome-editing tool. The plasmid pUC19 was used as the template for amplification of the ampicillin resistance gene. All strains and plasmids were obtained from an in-house repository.

The CRISPR/Cas9 editing vector pCRISPomyces-2 was constructed based on the method previously described by Cobb [46]. The sgRNA targeting *gluR* was cloned using Golden Gate assembly, and ~1 kb homologous arms flanking the gene were assembled by Gibson Assembly as described by Zeng [47]. The plasmid was transferred into *S. alboflavus* TD-1 by intergeneric conjugation using *E. coli* ET12567/pUZ8002, following the protocol outlined in *Practical Streptomyces Genetics*.

Genomic DNA from exconjugants was extracted, and PCR was performed to confirm integration. Primers gluR-F and gluR-R were used to verify replacement of *gluR* with the Amp gene, yielding an expected 778 bp PCR product. Another pair of primers, Amp-F and Amp-R, was used to verify that the Amp gene was present in exconjugants. The PCR amplification product was sequenced and compared with the GenBank reference sequence.

### 2.7. Assay for Inhibitory Effects of mVOCs on A. flavus

The inhibitory effects of mVOCs from *S. alboflavus* TD-1 and the *gluR* deletion mutant (TD-1 Δ*gluR*) on *A. flavus* were evaluated using a two-compartment Petri dish system to create a sealed co-culture environment. In the lower compartment, wheat bran cultures inoculated with either *S. alboflavus* TD-1 or TD-1 Δ*gluR* were incubated at 28 °C for 8 days to allow mVOC accumulation. The upper compartment contained 5 mL PDA inoculated in the center with a 6 mm plug of actively growing *A. flavus*. The plates were assembled vertically to form a sealed chamber (~75 mL headspace) and immediately sealed with two layers of Parafilm to prevent mVOC leakage. The assembly was incubated at 28 °C for 3 days. After incubation, the radial growth of *A. flavus* colonies was measured and compared between treatments. All experiments were conducted in triplicate. The inhibition rate was calculated as follows:

Inhibition (%) = (D_c_ − D_t_)/D_c_ × 100%

where D_c_ is the colony diameter of the control, and D_t_ is that of the treatment.

### 2.8. Statistical Analysis

Statistical analyses were performed using SPSS Statistics 22.0 (IBM, Armonk, NY, USA). Data are presented as mean ± standard deviation (SD). Differences among multiple groups were assessed via one-way analysis of variance (ANOVA). When significant effects were detected, post hoc comparisons were conducted using Fisher’s least significant difference (LSD) test. A p < 0.05 was considered statistically significant. Figures were prepared using Origin 9.0 (Origin Lab, Northampton, MA, USA).

## 3. Results

### 3.1. Effect of Glutamine on Antifungal Activity and mVOC Production in S. alboflavus TD-1

**Figure 1:**
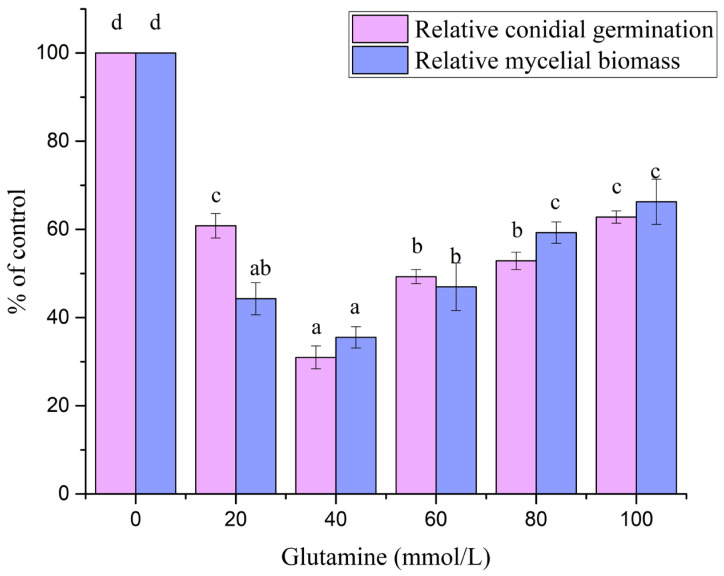
Effect of glutamine concentration on the antifungal activity of *S. alboflavus* TD-1 against *A. flavus*. Relative conidial germination and relative mycelial biomass were quantified after 3 days of co-incubation in a sealed dual-Petri dish system. Values are expressed as percentages of the 0 mM glutamine control (set to 100%). Bars represent the mean ± SD (*n* = 3). Different letters indicate significant differences among glutamine concentrations within the same parameter (one-way ANOVA followed by LSD test, *p* < 0.05).

All glutamine treatments significantly inhibited A. flavus relative to the 0 mM control, as reflected by reduced conidial germination and decreased dry mycelial biomass (*p* < 0.05; Figure 1). The strongest effect was observed at 40 mM glutamine, where relative conidial germination decreased to 31.0% of the control (=69.0% inhibition) and relative mycelial biomass decreased to 35.5% of the control (=64.5% inhibition). Increasing glutamine beyond 40 mM did not further improve inhibition (*p* > 0.05 compared with 40 mM), indicating a plateau in the growth-suppression effect under the tested conditions.

To determine whether the enhanced antifungal activity was associated with increased mVOC production, headspace VOCs were profiled using GC-MS from cultures grown in the same dual-Petri dish setup, but incubated for 8 days to ensure sufficient mVOC accumulation. Across all treatments, a total of 21 mVOCs were identified, spanning hydrocarbons, ketones, terpenoids, alcohols, ethers, and other compounds (Table 2).

GC-MS profiling identified 21 VOCs, including hydrocarbons, ketones, terpenoids, and ethers (Table 2). Notably, supplementation with 40 mM glutamine led to marked increases in four VOCs with reported antifungal activity: dimethyl trisulfide, *o*-anisidine, anisole, and 1,5-cyclooctadiene. Their abundances increased 1.55- to 10.56-fold compared with the control, with the largest increases noted for 1,5-cyclooctadiene (10.55-fold) and dimethyl trisulfide (6.17-fold) (Table 2).

### 3.2. Transcriptomic Response of S. alboflavus TD-1 to Glutamine Supplementation

Two sequencing libraries were prepared from the control group (CG) and the glutamine-supplemented group (EG) to examine the transcriptomic response of *S. alboflavus* TD-1 to glutamine addition. The total mRNA reads for CG and EG were 43,292,342 and 44,852,360, respectively. The raw reads had an average length of 380 bp, and total data output exceeding 6.5 Gb for each group. Quality assessment showed that more than 92% of bases had Q30 scores (>99.9% base call accuracy), and more than 96% achieved Q20 scores (>99% accuracy).

Differential gene expression analysis was performed using the DESeq2 package, applying a threshold of *q*-value < 0.05 and |log_2_FoldChange| > 1. A total of 283 differentially expressed genes (DEGs) were identified in the EG compared to CG, including 173 upregulated and 110 downregulated genes.

**Figure 2:**
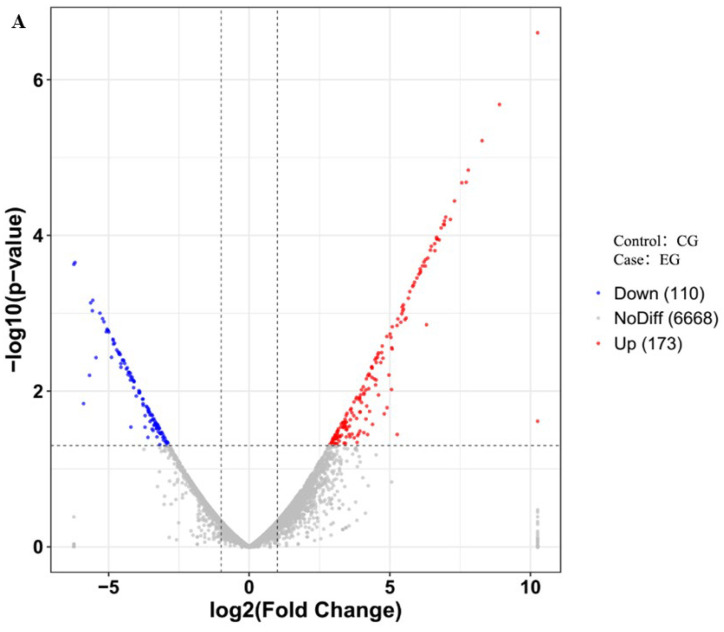

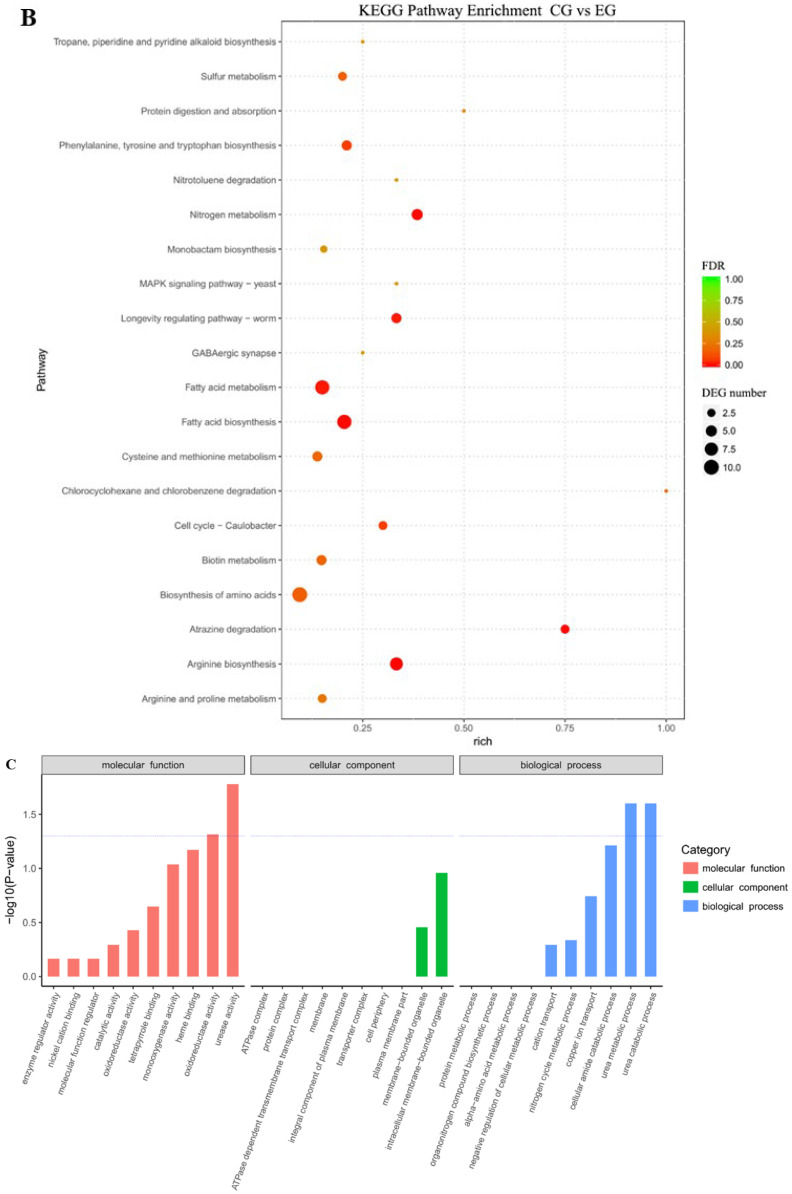
Transcriptomic analysis of *S. alboflavus* TD-1 under glutamine supplementation. (**A**) Volcano plot of differentially expressed genes. Red indicates upregulated genes, and blue indicates downregulated genes. (**B**) KEGG pathway analysis of DEGs. (**C**) GO functional analysis of enriched terms. The *x*-axis corresponds to the categories, and the *y*-axis corresponds to the mean expression value of −log_10_ (*p*-value) of enriched categories. EG: experimental group (40 mM glutamine); CG: control group (0 mM glutamine).

GO analysis showed significant enrichment in biological processes such as urease activity, oxidoreductase activity, urea metabolic process, and urea catabolic process (Figure 2A). KEGG pathway analysis revealed 20 significantly enriched pathways, including amino acid metabolism, nitrogen metabolism, atrazine degradation, sulfur metabolism, and fatty acid metabolism (Figure 2B).

Based on GO and KEGG annotations, several enriched pathways—particularly those involved in nitrogen metabolism, amino acid metabolism, fatty acid metabolism, and sulfur metabolism—have been reported to provide precursors for the biosynthesis of mVOCs, including nitrogen- or sulfur-containing volatiles as well as lipid-derived compounds. These associations suggest potential links between glutamine supplementation and modulation of metabolic routes that could influence mVOC biosynthesis (Table 3, Figure 2C). However, these relationships are inferred from pathway annotation rather than direct functional evidence, and further experimental validation is required to confirm their specific roles in mVOC production.

**Figure 3:**
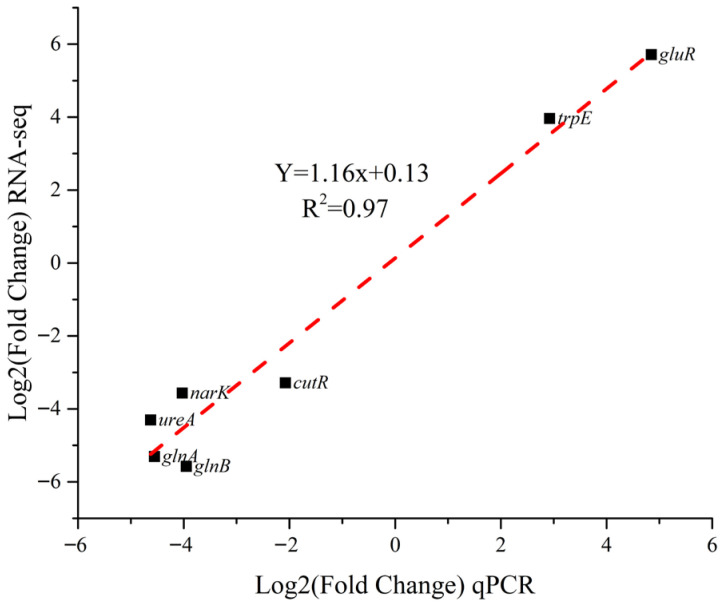
Correlation between RNA-Seq and qRT-PCR results for seven selected DEGs (*cutR*, *glnA*, *glnB*, *gluR*, *narK*, *trpE*, and *ureA*). Each point represents the log_2_(fold change) value of one gene. A strong positive correlation was observed between the two methods (R^2^ = 0.97, *p* < 0.01), confirming the reliability of the transcriptomic data. Log2 (EG/CG): Results are expressed as fold change (log_2_) of EG relative to CG; EG: experimental group (40 mM glutamine); CG: control group (0mM glutamine); each bar represents the mean of three replicates ± standard error.

To assess the reliability of the RNA-Seq data, seven DEGs (*cutR*, *glnA*, *glnB*, *gluR*, *narK*, *trpE*, and *ureA*) were randomly selected for validation by qPCR. The expression patterns of these genes were highly consistent with the RNA-Seq results (Figure 3, R^2^ = 0.97, *p* < 0.01). This strong correlation validated the robustness and accuracy of the transcriptomic analysis.

### 3.3. CRISPR/Cas9-Mediated Deletion of gluR

**Figure 4:**
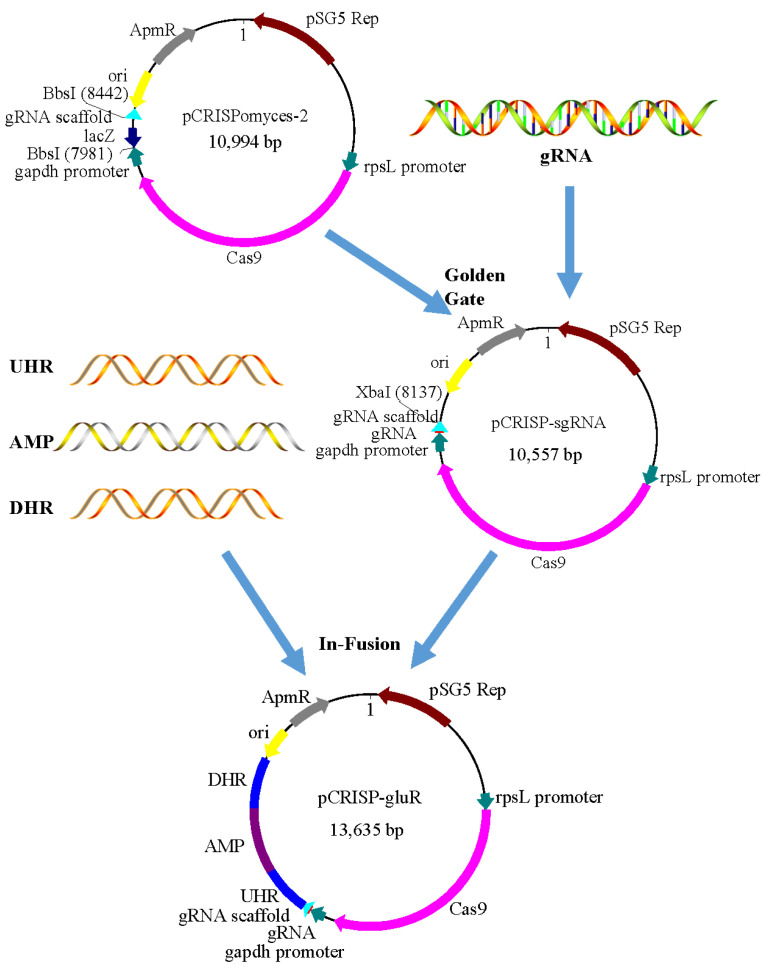
Workflow of CRISPR–Cas9-mediated construction of the *gluR* knockout mutant in *S. alboflavus* TD-1.

The CRISPR/Cas9 vector pCRISPomyces-2 was used to delete the *gluR* gene and generate the mutant strain TD-1 Δ*gluR* carrying the ampicillin resistance gene. The workflow of CRISPR–Cas9 *gluR* knockout is shown in Figure 4.

**Figure 5:**
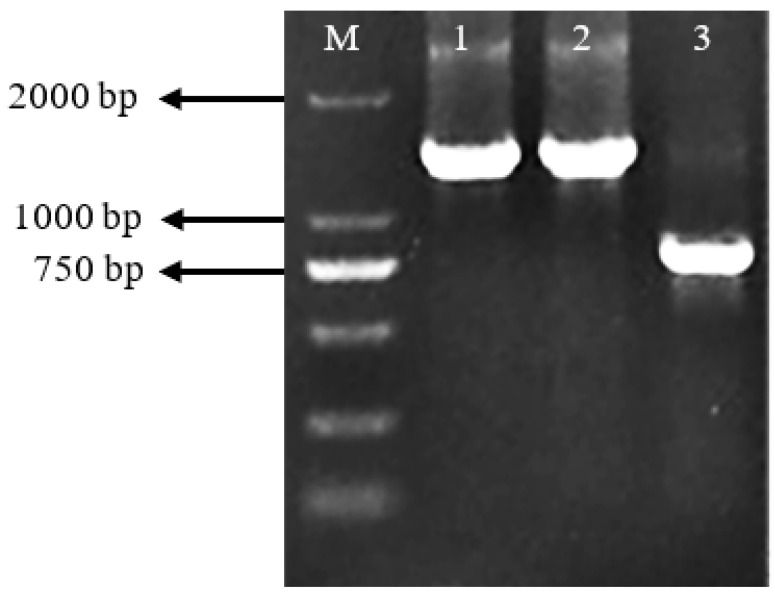
Electrophoresis analysis to identify the transformants after PCR. Primer gluR-F and gluR-R were used to confirm the sequence at the mutation site, with *S. alboflavus* TD-1 serving as a control. The 778 bp *gluR* fragment was amplified from *S. alboflavus* TD-1, whereas the 1394 bp (1227 bp of ampicillin gene and an extra 167 bp from the genome) amplicon was amplified from *S. alboflavus* TD-1 Δ*gluR*; M: DL2000 Maker; 1, 2: Gene knockout transformants of *S. alboflavus* TD-1 Δ*gluR*; 3: Gene transformants of *S. alboflavus* TD-1.

Exconjugants were verified by PCR using gluR-F/gluR-R and Amp-F/Amp-R primers. In the wild-type strain, a 778 bp fragment of *gluR* was amplified, while in the Δ*gluR* mutant, a 1394 bp fragment (1227 bp Amp gene + 167 bp flanking sequence) was obtained (Figure 5). DNA sequencing confirmed successful replacement of *gluR* with the *Amp* gene.

### 3.4. Effects of gluR Deletion on Phenotype, Antifungal Activity, and mVOC Profiles

**Figure 6:**
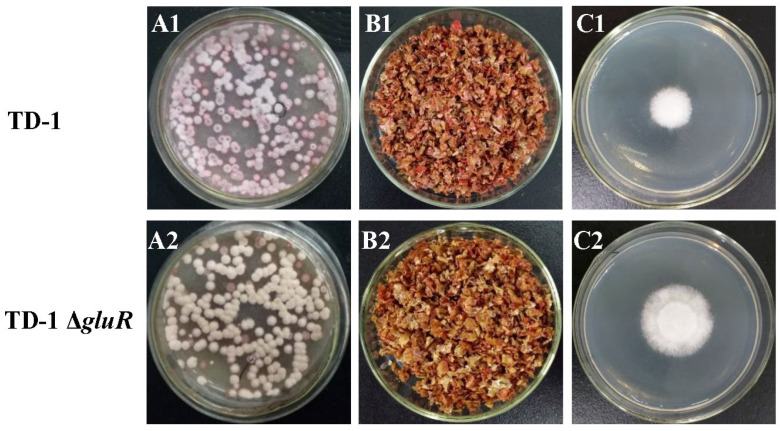
Inhibitory effect of mVOCs from *S. alboflavus* TD-1 and *S. alboflavus* TD-1 Δ*gluR* against *A. flavus*. (**A1**) *S. alboflavus* TD-1 was incubated at 28 °C for 8 days. (**A2**) *S. alboflavus TD-1* Δ*gluR* was incubated at 28 °C for 8 days. (**B1**) The lower plate containing different amounts of wheat bran culture of *S. alboflavus* TD-1. (**B2**) The lower plate containing different amounts of wheat bran culture of *S. alboflavus* TD-1 Δ*gluR*. (**C1**) The upper plate containing 5 mL of PDA inoculated within a 6 mm diameter *A. flavus* plug and exposed to mVOCs from *S. alboflavus* TD-1. (**C2**) The upper plate containing 5 mL of PDA inoculated within a 6 mm diameter *A. flavus* plug and exposed to mVOCs from *S. alboflavus* TD-1 Δ*gluR*.

Deletion of the *gluR* gene in *S. alboflavus* TD-1 resulted in noticeable phenotypic changes. The Δ*gluR* mutant displayed altered colony coloration, with reduced red pigment production and more vigorous aerial mycelium (Figure 6A1,A2). In contrast, the general morphology of the mycelial network remained comparable to that of the wild-type strain (Figure 6B1,B2).

Functionally, the inhibitory activity of mVOCs produced by the Δ*gluR* mutant against *A. flavus* was significantly reduced. The wild-type strain inhibited *A. flavus* growth by 68.96%, whereas inhibition by the Δ*gluR* mutant was only 50.57% (Figure 6C1,C2), highlighting the importance of *gluR* in regulating antifungal capacity.

GC-MS analysis further revealed substantial differences in mVOC composition between the two strains (Table 4). In the Δ*gluR* mutant, concentrations of sulfides, ethers, most terpenes, and ketones were markedly reduced, whereas hydrocarbons, aldehydes, and some terpenes were elevated relative to the wild-type strain. Importantly, five antifungal mVOCs—anisole, dimethyl trisulfide, *o*-anisidine, 1,5-cyclooctadiene, and β-pinene—were consistently lower in the mutant, together with other bioactive compounds such as D-limonene, 2-methylisoborneol, and isoledene.

Taken together, these results demonstrate that *gluR* deletion not only alters the colony phenotype of *S. alboflavus* TD-1 but also reduces its antifungal activity, primarily through downregulation of key antifungal mVOCs. This finding indicates that *gluR* plays a dual regulatory role in mVOC biosynthesis, exerting both positive and negative effects on metabolite production, with its positive regulation of compounds such as dimethyl trisulfide and *o*-anisidine being particularly critical for effective inhibition of *A. flavus*.

## 4. Discussion

*A. flavus* is a major postharvest concern because it readily contaminates cereals, nuts, and other commodities and can lead to aflatoxin accumulation, posing a direct food safety risk [17,48]. mVOCs produced by biocontrol microbes have therefore attracted growing interest as low-residue antifungal agents, particularly in enclosed or semi-enclosed settings such as storage and packaging systems [18,19]. A persistent practical challenge is that mVOC emission is highly sensitive to culture conditions, and is often difficult to reproduce across different media and environments. In this study, our results indicate that glutamine supplementation can be used as a simple lever to enhance the antifungal mVOCs of *S. alboflavus* TD-1 under the conditions tested. In addition, the results also suggest that GluR is involved in linking nutrient status to mVOC-mediated antagonism [20].

This enhancement is supported by consistent phenotypic outcomes. At 40 mM glutamine, TD-1 showed the strongest inhibition, suppressing *A. flavus* conidial germination by 69.0% and reducing dry mycelial biomass by 64.5% relative to the control. Together, these readouts reflect both early developmental arrest and reduced vegetative accumulation, which are directly relevant to limiting fungal establishment during storage and handling.

An important feature of the response was its non-linearity. Antifungal performance peaked at 40 mM glutamine and did not improve further at concentrations of 60 mM and above. This pattern is consistent with the view that glutamine acts not only as a nitrogen source but also as a regulatory metabolite capable of reshaping secondary metabolism [16,36]. From an application standpoint, the finding argues for dose optimization rather than simple nutrient maximization, and it raises the possibility that excessive nitrogen signaling may shift metabolism away from the mVOC subset most responsible for antifungal activity. At least two explanations are plausible. Nitrogen-sufficiency signaling could trigger feedback regulation that alters carbon–nitrogen allocation and limits precursor supply for particular mVOC pathways [49]. In addition, higher glutamine levels may alter the local physicochemical environment in ways that broadly reprogram secondary metabolism. These possibilities are not mutually exclusive, and future work that combines glutamine gradients with intracellular metabolite measurements and absolute mVOC quantification should help clarify whether the decline at higher concentrations reflects precursor limitation, physiological shifts, or both [50].

mVOC profiling provides a useful bridge between glutamine supplementation and enhanced antifungal activity. TD-1 produced a defined mVOC repertoire, and glutamine mainly altered the relative abundance of multiple compounds rather than generating an entirely new mVOC profile. It is important to note that the changes reported here are relative and derived from GC-MS peak-area comparisons between experimental and control conditions (log2 (EG/CG)), rather than absolute headspace concentrations. Under treatment with 40 mM glutamine, several compounds increased markedly, with the largest changes reaching approximately 12.47-fold. Notable increases were observed for 1,4-dimethyladamantane, 1,5-cyclooctadiene, and azulene, along with other hydrocarbons and aromatics. Glutamine also elevated compounds that have been repeatedly associated with antimicrobial activity in microbial volatile compounds, including dimethyl trisulfide as well as anisole and o-anisidine [26,51].

mVOCs typically act as blends, and antifungal outcomes often reflect mixture effects rather than the action of a single compound. The improved antagonism observed here is therefore likely to result from both the increased abundance of inhibitory mVOCs reported in the literature and a shift in the overall blend that changes how the mixture interacts with the target fungus. Nevertheless, these mixture-level mechanisms remain inferential at present. The most direct way to strengthen causal attribution, particularly in a food context, is to test candidate mVOCs at headspace-relevant levels and then examine whether reconstructed mixtures recapitulate the activity of the natural blend. Linking inhibition to absolute emissions, rather than relative changes alone, will also be essential [19,20].

Transcriptomic data provide a broader metabolic rationale for the observed volatile compounds remodeling. Glutamine induced 283 differentially expressed genes, with enrichment in pathways related to amino acid metabolism, sulfur metabolism, fatty acid metabolism, and transport or regulatory functions. Several genes associated with nitrogen assimilation and urea-related metabolism were downregulated, consistent with a nitrogen-sufficiency state that may redistribute flux. The strong induction of sulfonate utilization and transport genes, such as *ssuC* and *ssuD*, offers a plausible connection to increased sulfur volatiles including dimethyl trisulfide. Changes in fatty acid metabolism genes, including *fabH* and *fabG*, are also consistent with shifts in precursors that contribute to hydrocarbon- and ketone-type mVOCs [34,52]. Although transcriptomics cannot by itself assign each mVOC to a specific biosynthetic route, it highlights sulfur handling and lipid metabolism as high-priority nodes for targeted validation [30,49].

Our data further support a role of GluR in glutamine-responsive regulation of antifungal mVOC output. Glutamine increased *gluR* expression, and deletion of *gluR* attenuated mVOC-mediated antagonism, with inhibition decreasing from 68.96% in the wild-type strain to 50.57% in Δ*gluR*. GC-MS comparisons were consistent with this phenotype and indicated broad mVOC shifts in the mutant, including reduced levels of candidate antifungal compounds such as dimethyl trisulfide and anisole. These findings support a working model in which GluR contributes to a nutrient-responsive regulatory program that promotes production and/or emission of an antifungal mVOC blend [37]. In addition, the mechanistic boundary should be stated clearly. The Δ*gluR* phenotype may reflect direct regulatory control of mVOC-associated pathways, but it could also arise indirectly through altered growth or emission capacity. Complementation of Δ*gluR*, normalization of mVOC emissions to biomass, and identification of downstream targets would provide the most convincing confirmation [52].

From the perspective of foods, translating these findings to real commodities requires careful attention to matrix and headspace effects [14,38]. mVOC concentrations and composition in sealed Petri dish systems can differ substantially from those in grain bulks or packaged foods because ventilation, adsorption to matrices, and humidity-dependent partitioning all influence effective exposure. In addition, some mVOCs, particularly sulfur-containing and aromatic compounds, may affect sensory properties [20,27]. Any practical storage or packaging application should evaluate not only antifungal efficacy but also sensory acceptability, regulatory constraints, and safety, and should directly quantify aflatoxin outcomes where relevant. Another practical question is how reliably glutamine can steer mVOC output in complex substrates, where it may be consumed by other microbes or interact with the commodity matrix. Moving forward, it will be important to quantify key mVOCs absolutely across glutamine levels and then test performance directly in commodity systems under storage-relevant temperature and humidity [23,38]. Importantly, inhibition of *A. flavus* growth does not necessarily predict aflatoxin outcomes because aflatoxin biosynthesis can be uncoupled from biomass accumulation, and stress conditions may suppress growth while maintaining or even increasing toxin production. In this study, we did not quantify aflatoxins under VOC exposure; therefore, our conclusions are limited to growth suppression and the glutamine/GluR-dependent modulation of mVOC profiles. Direct measurement of aflatoxins in mVOC-exposed cultures and in commodity-relevant storage systems will be necessary to determine whether the observed growth inhibition is accompanied by decreased aflatoxin production/accumulation under mVOC exposure.

Taken together, these results show that glutamine can modulate the TD-1 volatile compounds in a way that improves antifungal performance under controlled conditions and that GluR is implicated as a regulatory node linking nutrient sensing to mVOC-mediated antagonism. The work provides a mechanistic starting point for improving the robustness of mVOC-based suppression of *A. flavus* and outlines the practical steps needed to translate laboratory observations into postharvest applications.

## 5. Conclusions

This study demonstrated that glutamine has a significant influence on mVOC production in *S. alboflavus* TD-1. The *gluR* gene was found to positively regulate the biosynthesis of sulfides, ethers, most terpenes, and ketones, while exerting negative regulatory effects on hydrocarbons, aldehydes, and certain terpenes. Transcriptomic analysis combined with the *gluR* deletion mutant revealed that glutamine regulates amino acid and nitrogen metabolism through the GluR two-component system, thereby modulating mVOC biosynthesis and enhancing the biocontrol efficacy of *S. alboflavus* TD-1 against *A. flavus*. Taken together, these findings highlight the dual role of glutamine as a metabolic substrate and regulatory signal, linking nutrient sensing to secondary metabolism and antifungal activity. Beyond advancing our mechanistic understanding, this work offers promising directions for developing glutamine-enhanced mVOCs as a sustainable strategy to suppress *A. flavus* growth, with aflatoxin outcomes requiring direct validation in commodity-relevant systems. Nevertheless, further research is needed to validate these findings under in vivo and field conditions, to optimize application methods, and to assess ecological safety and scalability.

## Figures and Tables

**Table 1:** Primers used for RT-PCR in this study.

Gene	Primer Sequence (5′-3′)
*16 S rRNA*	Forward primer 5′-CGACGCAACGCGAAGAACCT-3′
	Reverse primer 5′-GACGACAGCCATGCACCACC-3′
*trpE*	Forward primer 5′-CCCGACGAGGAGGAGAACC-3′
	Reverse primer 5′-GATGTCGTTGCGGCACAGG-3′
*glnA*	Forward primer 5′-CTGCCGATCTGGGGTTTCG-3′
	Reverse primer 5′-TCCAGGACCTCGCACAGGA-3′
*glnB*	Forward primer 5′-GATCCGCATCGAGGTGCTC-3′
	Reverse primer 5′-TTGCCGTCGCCGATCTTGC-3′
*gluR*	Forward primer 5′-ATCCACGGCCTCAACCTCG-3′
	Reverse primer 5′-GTGTCGGCGGACTTCTTCG-3′
*cutR*	Forward primer 5′-CCCAAGCCCTTCGCGTTCA-3′
	Reverse primer 5′-TCCTTGCCGTCGCGGAACA-3′
*nasA*	Forward primer 5′-CGTGAACACGGGCAGAAGG-3′
	Reverse primer 5′-GCGTCGAGAAGCTCGTAGGC-3′
*narK*	Forward primer 5′-AGCCCGCTGAGCACGAAGA-3′
	Reverse primer 5′-CCGCATCACGCTGTGGAACT-3′
*sgRNA*	Forward primer 5′-GCGTCTACGGGCACCTTACC-3′
	Reverse primer 5′-TCGCCACCTCTGACTTGAGC-3′
*UHR*	Forward primer 5′-GCGTTTTTTATCTAGATCGTCGAAGAGCATCACCT-3′
	Reverse primer 5′-GGTGATGACGGTGAAGCAGTCTCATACGGTCTCCCT-3′
*DHR*	Forward primer 5′-GTTCCACTGAGCGTCCTGCACATGCCCGCCCTGAT-3′
	Reverse primer 5′-GGTTCCTGGCCTCTAGAGCGATCTCCTCGTTGCCCTC-3′
*AMP*	Forward primer 5′-ACCGTATGAGACTGCTTCACCGTCATCACCGAAAC-3′
	Reverse primer 5′-GGCGGGCATGTGCAGGACGCTCAGTGGAACGAAAA-3′

**Table 2:** Effect of glutamine on mVOCs from *S. alboflavus* TD-1 incubated at 28 °C for 8 days.

Compounds	RT	Log2 (EG/CG)
Hydrocarbons		
3-Heptene *	13.21	−0.49
Cycloheptane *	14.16	−0.24
Nonane *	15.25	−0.30
2-Decene	16.70	−0.11
4-Decene *	19.83	−0.46
1,3-Cyclopentadiene	12.12	0.09
1,5-Cyclooctadiene *	21.63	3.40
1H-Indene *	22.10	3.22
1,4-Dimethyladamantane *	22.65	3.64
Azulene *	30.05	3.36
Naphthalene *	30.38	1.81
Ketones		
2-Decanone *	21.02	0.93
Terpenoids		
beta-Pinene *	11.84	−0.30
2-Methyl-2-bornene	13.70	0.24
2-Methylisoborneol *	20.50	1.20
beta-Copaene	30.90	0.12
Alcohols		
4a(2H)-Naphthalenol *	28.61	3.34
Ethers		
Anisole *	9.54	0.84
Others		
Dimethyl trisulfide *	11.56	2.65
*o*-Anisidine *	19.88	0.63
2H-3,9a-Methano-1-benzoxepin *	30.31	3.05

* Denotes a significant difference between the treatment group and the control group, *p* < 0.05. Key: RT: retention time in minutes; EG: experimental group (40 mM glutamine); CG: control group (0 mM glutamine).

**Table 3:** Details on differentially expressed genes involved in mVOC synthesis.

Gene ID	Gene Name	Log2 (EG/CG)	*p*-Value	Function
Nitrogen metabolism				
gene1147	*ureC*	−3.35	2.44 × 10^−2^	Urease subunit alpha
gene1148	*ureB*	−5.89	1.45 × 10^−2^	Urease subunit beta
gene1149	*ureA*	−4.31	6.14 × 10^−3^	Urease subunit gamma
gene2353	*glnA*	−5.31	9.99 × 10^−4^	Glutamine synthetase
gene2671	*nasA*	−5.56	6.81 × 10^−4^	Nitrite reductase
gene3194	*narK*	−3.57	1.80 × 10^−2^	Nitrate/nitrite transporter
gene6084	*glnB*	−5.58	9.27 × 10^−4^	Nitrogen regulatory protein
Amino acid metabolism				
gene469	*slcC*	4.18	1.55 × 10^−2^	Glyoxylate reductase
gene3047	*trpE*	3.96	1.12 × 10^−2^	Anthranilate synthase
gene3048	*trpG*	3.40	2.85 × 10^−2^	Anthranilate synthase
gene4517	*speE*	3.14	3.51 × 10^−2^	Spermidine synthase
gene6372	*aspC*	5.81	4.52 × 10^−4^	Aspartate aminotransferase
gene7223	*argD*	2.99	4.09 × 10^−2^	Acetylornithine aminotransferase
Fatty acid metabolism				
gene3964	*fabH*	5.07	2.78 × 10^−3^	3-Oxoacyl-ACP synthase
gene3965	*fabG*	4.49	8.32 × 10^−3^	Hypothetical protein
gene381	*curA*	−4.09	7.54 × 10^−3^	Hypothetical protein
gene383	*fabG*	−4.29	5.72 × 10^−5^	Hypothetical protein
gene384	*fabH*	−4.66	3.13 × 10^−3^	3-Oxoacyl-ACP synthase
Sulfur metabolism				
gene6566	*sir*	2.97	4.11 × 10^−2^	Sulfite reductase
gene7899	*ssuC*	6.94	6.52 × 10^−5^	ABC transporter permease
gene7975	*ssuD*	6.17	2.46 × 10^−4^	Alkanesulfonate monooxygenase
Transcriptional regulator				
gene1755	*tctB*	3.45	3.87 × 10^−2^	Integral membrane protein
gene1895	*tetR*	4.21	3.60 × 10^−2^	TetR family transcriptional regulator
gene3049	*gabR*	3.06	3.71 × 10^−2^	GntR family transcriptional regulator
gene6252	*gluR*	5.71	5.27 × 10^−4^	Sensory transcriptional regulator
gene6339	*cutR*	−3.29	2.66 × 10^−2^	Transcriptional regulatory protein

Significance: The threshold value of significant differential expression was a fold change >2 and corrected *p*-value < 0.05. Log2 (EG/CG): Results are expressed as fold change (log2) of EG relative to CG; EG: experimental group (40 mM glutamine); CG: control group (0 mM glutamine).

**Table 4:** Differential mVOCs between *S. alboflavus* TD-1 Δ*gluR* and the wild-type strain.

Compounds	RT	Log2 (EG/CG)
Hydrocarbons		
Hexane *	5.40	0.52
Heptane	13.45	0.02
Nonane *	15.25	0.38
2-Undecene *	16.10	0.52
Decane *	17.07	0.48
Octane *	17.60	0.59
Dodecane	21.01	0.12
1,4-Dimethyladamantane	22.65	0.05
Azulene	30.05	0.07
Naphthalene *	30.38	0.26
2,4-Dimethyl-1-heptene *	5.15	−0.84
1,3-Cyclopentadiene *	12.12	−1.01
1,5-Cyclooctadiene	21.63	−0.09
1H-Indene	22.10	−0.03
Cyclohexane	23.01	−0.08
Heneicosane	28.82	−0.02
Ketones		
2-Decanone	21.01	0.15
2-Hexanone *	5.01	−0.86
2-Heptanone *	8.53	−0.30
Alcohols		
1-Hexanol *	7.83	−0.61
4a(2H)-Naphthalenol *	28.61	−0.71
Aldehydes		
Heptanal *	8.70	1.03
Benzaldehyde *	11.21	0.33
Terpenoids		
Cedrene	29.70	0.18
gamma-Muurolene	30.69	0.11
beta-Copaene	30.90	0.10
beta-Pinene *	11.83	−0.83
2-Methyl-2-bornene *	13.70	−0.96
D-Limonene *	14.13	−0.29
2-Methylisoborneol	20.50	−0.20
Isoledene	32.18	−0.03
alpha-Muurolene *	32.75	−0.26
Ethers		
Anisole *	9.51	−0.63
Others		
Furan	12.56	0.05
2H-3,9a-Methano-1-benzoxepin *	31.93	0.69
Dimethyl trisulfide *	11.56	−0.63
*o*-Anisidine	19.88	−0.19

* Denotes significant difference between TD-1 Δ*gluR* and the wild-type strain, *p* < 0.05. Key: RT: retention time (min); EG: Δ*gluR*; CG: wild type (TD-1).

## Data Availability

The original contributions presented in the study are included in the article, further inquiries can be directed to the corresponding authors.
